# Explore the Bereavement Needs of Families of Children With Cancer From the Perspective of Health Caregivers: A Qualitative Study

**DOI:** 10.3389/fpsyg.2021.750838

**Published:** 2021-10-18

**Authors:** Maryam Pakseresht, Maryam Rassouli, Nahid Rejeh, Shahnaz Rostami, Salman Barasteh, Shahram Molavynejad

**Affiliations:** ^1^School of Nursing and Midwifery, Ilam University of Medical Sciences, Ilam, Iran; ^2^Cancer Research Center, Shahid Beheshti University of Medical Sciences, Tehran, Iran; ^3^Elderly Care Research Center, Faculty of Nursing and Midwifery Shahed University, Tehran, Iran; ^4^Nursing Care Research Center in Chronic Diseases, School of Nursing and Midwifery, Ahvaz Jundishapur University of Medical Sciences, Ahvaz, Iran; ^5^Health Management Research Center, Nursing Faculty, Baqiyatallah University of Medical Sciences, Tehran, Iran

**Keywords:** pediatric cancer, death, bereavement, family, grief, care, self-control

## Abstract

**Introduction:** The experience of bereavement is associated with severe physical, psychological, social and spiritual reactions in the parents of children with cancer. Because of that, the families of these children need to receive bereavement services. The aim of this study was to explore the bereavement needs of families of children with cancer from the perspective of health caregiver as people who have a close relationship with the child and the family.

**Methods:** This qualitative descriptive study design in. In total 15 semi-structured interviews were conducted using a purposive sampling in 2018. Interviews were recorded and transcribed and conventional content analysis was used to analysis the data. The Trustworthiness of the data were assessed according to the criteria of Lincoln and Guba.

**Results:** From the data analysis, needs of the bereaved family were categorized in three dimension including “achieving peace,” “Abandoned family access to care,” and “continuing care.” The category of “achieving peace” includes spiritual and existential support, companionship with the family, contact with other bereaved families, support in passing and accepting the bereaved and continuing empathetic communication with the family, the category “Abandoned family access to care” includes the promotion of family self-control, awareness of end-of-life care to the family, and the category of “continuing care,” includes formal and informal family care and individualized care.

**Conclusion:** It is necessary for the care team to pay special focus to family considering the needs of the family about the death of the patient and the challenges of the family bereavement period. It is recommended that members of the health care team should be trained in assessing family needs, identifying risks of adverse outcomes, continuing care, and providing resources during bereavement. The needs of the bereaved family should also be addressed in their care plan.

## Introduction

Pediatric death by cancer is a serious problem worldwide, with more than 413,000 death caused in 2020 (The American Childhood Cancer Organization, 2021). Annually up to 2,000 children under 15 years are diagnosed with cancer in Iran (Pakpour et al., 2019). Cancer is the second leading cause of death in children under 14 in Iran. It causes about 4% of deaths of children under 5 and 13% of deaths of children aged 5–10 years (Kashani, 2012).

Cancer causes physical, psychological, social, economic, and emotional reactions for child and family (Zarit, 2004; Lu et al., 2009; Valizadeh et al., 2014) that children's reactions to these stresses include physical problems, depression, anxiety, fear, behavioral, and communication problems, decreased self-esteem and social interactions, and retrograde behaviors that are affected by the child's developmental stages and adaptability, duration of stress, and Its intensity (Kirsch et al., 2003; Yahia et al., 2015) and imposes a heavy burden of care on the family (Zaider and Kissane, 2010).

The death of a child due to cancer is a devastating event that leads to prolonged bereavement (Snaman et al., 2020), complex traumatic reactions to bereavement (Gilmer et al., 2012), dissolution of the attachment relationship with the child, and feeling guilty. Grief is defined as an internal experience in reaction to the loss of something loved and valued (Näppä et al., 2016). Parental grief is prolonged 5–7 and can intensify during significant periods (e.g., holidays), a concept known as “regrief” (Gilmer et al., 2012).

Bereavement is mourning with a strong sense of loss and sadness that occurs with death and is a process in which life continues without the presence of the person (Pakseresht et al., 2018). These severe and prolonged reactions to grief are common among family members but sometimes can be debilitating (Lichtenthal et al., 2015). All people grieve differently; some need professional help while others are resilient in their loss and do not require special interventions (Näppä et al., 2016).

Numerous studies have examined the psychological challenges such as depression and anxiety for parents with pediatric cancer (Kreicbergs et al., 2004; Kim et al., 2013), grief (Lannen et al., 2008), existential anxiety, identity, and spiritual challenges (Lichtenthal et al., 2015), guilt (Surkan et al., 2006), post-traumatic stress disorder, negative impact on copping, adverse effects on health, social interactions, and high levels of psychological distress that often require professional assessment and assistance (Gilmer et al., 2012; Kim et al., 2013). Parents who have lost a child to cancer, they feel that if they could prevent cancer or death, they often experience self-blame and guilt (Eslahkar et al., 2019). However, despite this misery, most grieving individuals do not develop mental and or physical complications. A minor number of significant other in the bereavement period show increased risk for hospitalization and death as well as depression, mental illness, and substance abuse (Näppä et al., 2016).

The presence of such symptoms, as well as the disease and its treatments side-effects, the high cost of treatment, psychological and social consequences of the disease of the child and family, require comprehensive care in the form of supportive care. This type of care supports the family and those around the dying or deceased child (Pakseresht et al., 2018). Communication, continuity of care by caregivers, as well as child's physical and mental symptoms management, which are very important for parents in the later stages of a child's life, are aspects of parental care during the bereavement period before the child's death (van der Geest et al., 2016). Family support at the end of a child's life and after death contributes to the bereavement process in families, as well as providing an opportunity for the family to share their feelings with those who understand them (Pakseresht et al., 2018). Attention to, emotional, physical, environmental and psychosocial needs are the criteria considered during family bereavement (Suttle et al., 2017). Despite this need, care for bereaved parents did not exist widely 10 years ago, and pediatric providers might not have knowledge, even when bereavement care programs exist (Spraker-Perlman et al., 2021).

According to a study, families who do not receive professional bereavement care live in a state of prolong uncertainty and anxiety. However, in addition to complex responses to loss, many parents have responded to this loss in constructive ways (Eslahkar et al., 2019). Parental demographics, such as age, gender, race, and ethnicity, affect the grief experience and bereavement outcomes (Snaman et al., 2020). One study found that parents over 30 years old showed better psychosocial adjustment than younger parents following the death of a child from cancer (Morrow et al., 1981). Reynolds et al. argued that grief and depression are normal emotions in bereavement, but that symptoms of grief resolve more slowly than medically treated depression (Reynolds et al., 2004).

Bereaved parents have identified a need for improved bereavement support, emphasizing the important role that the healthcare team and medical institution serve in their grief journey. Despite this need, care for bereaved parents did not exist widely 10 years ago, and pediatric providers might not have knowledge, even when bereavement care programs exist (Spraker-Perlman et al., 2021). There is a wide variety of bereavement programs where services can include a range of telephone or letter calling programs, grief support groups (siblings or parents), or meetings with professionals to discuss the death of a child or be the results of the autopsy (Widger et al., 2012; Yahia et al., 2015). Bereavement groups are believed to be beneficial as preventive interventions from social and economic standpoints. Participation is likely to be more acceptable and less threatening to potential recipients than professional interventions linked to psychiatry. Costs can be low, since groups are usually led by staff or volunteers rather than mental health professionals (Näppä et al., 2016). An experience not often discussed among providers is the personal feelings of grief that may come when a patient dies. Although providers of all disciplines have varied encouragement in engaging with the experience that may be personally felt when caring for those who are at end of life, the nurturing of individual self-care can be of benefit in regards to tenability and viability within such work (Jonas et al., 2018).

Among the developed countries, Britain has standards of bereavement care, published in 2001 (Kirsch et al., 2003; B. S. A. a and Care, 2013). In Iran, a special, targeted, and codified care program is not designed for a caring family bereavement, and families are practically abandoned after the death of a child (Rassouli and Sajjadi, 2014). The only cases observed are limited counseling to families after the death of a child in some centers (Pakseresht et al., 2018), including the Sherwin Charity, which provides bereavement care for the family as a group therapy by volunteer psychologists, and Due to the lack of sufficient evidence in assessing the need for these services, the country's policymakers are not aware of the necessity and priority of such services (Knapp, 2009).

Since one of the best ways to improve the quality of care is to identify the needs of the patient and family from different dimensions, examining the current state of bereavement care in the country from the perspective of nurses who have the most contact with the patient and family can be a step toward development of this type of care in medical centers.

Awareness of the needs of the child and family during bereavement provides a unique care plan for each family and prevents wastage of time and money following unscheduled care. Therefore, the present study was conducted to explore the bereavement needs of families of children with cancer from the perspective of nurses.

## Methods

### Design and Setting

In this present qualitative conventional content analysis 15 nurses were selected based on purposive sampling. This approach is used in situations where there has been previous research on a phenomenon but it needs further description (Potter and Levine-Donnerstein, 1999). Participants were selected from pediatric oncology ward of Mofid Hospital in Tehran (As a referral center from all regions of the country) and outpatient clinic of Shafa Hospital of in Ahvaz (Table 1). The research environment was the natural areas of employment or care in which study participants were routinely present.

**Table 1:** Demographic characteristics of care providers.

**No**	**Sex**	**Age (year)**	**Education (level)**	**Work experience (Years)**	**Interview time (Minutes)**
1	Female	32	B.A.	3	45
2	Female	35	M.A.	2	27
3	Female	28	B.A.	2	31
4	Female	24	B.A.	3	26
5	Female	23	B.A.	2	15
6	Female	38	M.A.	9	17
7	male	32	B.A.	3	15
8	Female	27	M.A.	5	19
9	Female	42	B.A.	15	24
10	male	29	B.A.	4	25
11	Female	30	B.A.	3	24
12	Female	40	B.A.	14	18
13	Female	38	B.A.	10	33
14	male	33	B.A.	10	37
15	Female	36	B.A.	12	21

### Participants and Sampling

These were the 15 official nurses in the pediatric cancer ward who had the most contact with the bereaved families and were selected based on purposive sampling. One of them was the headers. In the study of Spraker-Perlman et al. (2021), 13 mourning parents participated in the study, which despite the small number of participants, rich results were obtained in the study (Spraker-Perlman et al., 2021). The inclusion criteria was having experience in pediatric cancer wards for at least two year, Being formal and willingness to participate in study. Exclusion criteria included reluctance to interview and its continuation, failure to deal with the bereaved families of children with cancer. The first participants were a 32-year-old nurse who had 3 years of experience in the pediatric hematology department of Mofid Hospital in Tehran. This nurse had a close relationship with children with cancer and their families and provided complete and comprehensive information about patients and families to the researcher and was eager to conduct the interview.

The reason for choosing nurses instead of bereaved parents was that the nurses were in contact with families who had a child with cancer at the end of life or all of bereaved parents went to the hospital. So they could identify different needs in different families. While a mourning family could only share their special needs with us. Another reason for not choosing breaved parents was the lack of appropriate mental conditions for the interview. Interviews with nurses were able to gain a professional perspective on the needs of bereaved families.

### Data Collection

Semi-structured and face-to-face interviews were conducted by the first author in 2018. The first author was a doctoral student in nursing who had learned the method of interviewing during her studies from professors and the research team consisted of thesis supervisors and consultants who had sufficient experience in interview analysis. A guide to interview questions was prepared prior to the interview to gather comprehensive information. The interviews lasted between 15 and 45 min. All participants in this study were interviewed only once. Before conducting each interview, the researcher, by attending the research environment, while introducing himself/herself and explaining the nature and main purpose of the research, has identified the appropriate participant and while receiving written consent by specifying its provisions, the appropriate time and place agreed upon by the parties were determined based on the desire and preference of the participant. For the interview, the researcher introduced himself while explaining the main purpose of the research. Then the main question of the research was asked; “What is your experience in caring for families who have a child with cancer at the end of life or have lost their child?” What do you think are the needs of bereaved families? Probing questions then arose from experience of participants e.g., “What did you do as a health caregiver?” “Explain the current process of providing care for bereaved families” Was asked. Then the participant was asked to comment if they had any other points or questions. Interviews continued until data saturation. In this study, the needs of bereaved families at the end of child's life and also after the child's death were examined.

### Data Analysis

In order to analyze the qualitative data, conventional content analysis was used. This method was proposed in 2005 by Hsieh and Shannon (2005). This approach is used in situations where there has been previous research on a phenomenon but it needs further description (Potter and Levine-Donnerstein, 1999). In the present study, the entire text of the interview was read several times until a deep understanding was obtained. Then parts of the text were identified, marked, and turned into the smallest significant units (code) based on the researcher's initial perception. In the next step, the parts that were marked were encrypted based on predefined codes. Each part of the text that did not fit into this initial encryption was given a new encrypt. The author is inspired by other subject-related studies for coding. For this purpose, first, the text of the files was entered into Word software and then carefully read several times while key phrases and sentences were underlined. Then the codes (the main opinion of the participants) were extracted. The first level of the coding process began with identifying the meaning units, e.g., words, phrases, themes, and sentences that have specific meanings. Then the main concepts in each unit of analysis were called semantic units (Table 2).

**Table 2:** Conventional content analysis.

** 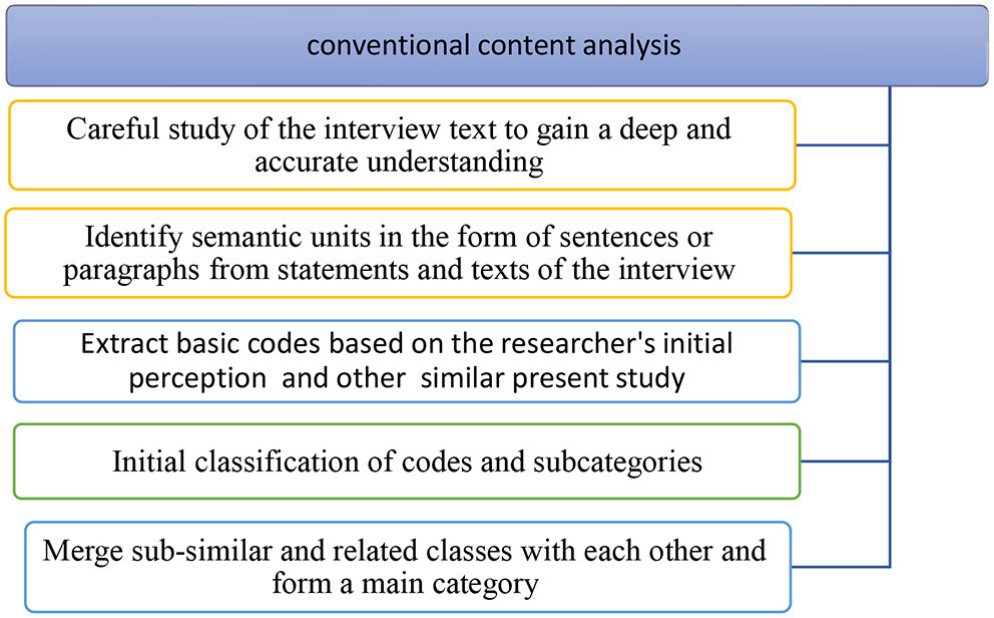 **

### Rigor and Trustworthiness

The Trustworthiness of the data were assessed according to the criteria of Lincoln and Guba (1985). These criteria including credibility, confirmability, dependability and transferability. In this study, long engagement with data was done by spending enough time to collect and analyze data, also review the data by supervisors and consultants and was used. Furthermore, the data were carefully examined by an external observer. In order to achieve the confirmability, all stages of the research, especially the data analysis in all directions were recorded in detail so that if another researcher wants to continue research in this field can easily understand. The type of research and the characteristics of the participants were well described in order to make a good judgment about the transferability of the readers.

### Ethical Considerations

The present study is part of the doctoral dissertation of the first author of the study, which has been approved by the ethics committee of Ahvaz Jundishapur University of Medical Sciences with No.IR.AJUMS.REC.1394.764. Interviews and audio recordings were conducted after participants' awareness of the purpose of the study and informed consent. Participants were also reassured that the recorded information would remain confidential, and it was announced that they could be excluded from the study at any time “without any penalty of sorts.”

## Results

A total of 400 codes were extracted from the interview analysis. The coding process was a continuous comparative analysis and duplicate codes was eliminated and similar codes was merged. Three categories named “achieving peace,” “abandoned family access to care,” and “continuity of care” and 9 subcategories were extracted (Table 3).

**Table 3:** Categories and subcategories.

**Category**	**Subcategory**
Achieving peace	Spiritual and existential support
	Companionship with the family
	Contact with other bereaved families
	Support in passing and accepting the bereaved
	Continuing empathetic communication with the family
Abandoned family access to care	The promotion of family self-control
	Awareness of end-of-life care to the family
continuing care	Formal and informal family care
	Individualized care

### Category 1: Achieving Peace

Although the health care team provides information and advice to the family, they ultimately prefer to choose options that provide peace. Participants considered the importance of providing peace for the family during the bereavement process. Subcategories of this category included “Spiritual and Existential Support,” “companionship with the family,” “Contact with other bereaved Families,” “Support in passing and accepting bereaved” and “continuous empathetic communication with the family” that they were considered soothing by some of the participants.

#### Spiritual and Existential Support

Based on participants' experience, spiritual and existential support has an effect on the sense of hope, value, purpose in connecting with others, and connecting to greater power in the child and family. “.Some people deny the illness of their child or family member due to the thought that it is a punishment from God. Sometimes their beliefs become so weak that they do not believe in anything. When you want to convince them that this is not the case, your work is a heavy burden.” (Nurse 1).

“One of the mothers of a child with cancer in the late stages of midnight said loudly, ‘God, you say you are more compassionate than my mother, so why?' A mother does not want her child to see her like this. so how do you want my child to die?” (Nurse 5).

“In my opinion, the existence of a psychologist, religious counselor, clergyman in these areas where mortality is very high and very effective. When you yourself have a strong belief in this field, you talk to the patient who comes to you and you will realize how calm your patient is…. There were families whose patients were on the verge of death. We easily persuaded him to sit on his head and pray for him. Easily accepted” (Nurse 9).

#### Companionship With the Family

Families deserve to be accompanied during the bereavement period and should be allowed to talk about their grief. Sharing emotions during bereavement with a friend or loved one may not help because people often try to comfort and the bereaved person stops sharing emotions. Almost all participants in this study emphasized the effect of health caregivers' behavior and their companionship on increasing or decreasing family morale. In the participants' view, accompanying and talking to the care team is more effective than accompanying those around them during the bereavement period.

“Let's give the patients' companion more time and opportunity to see the patient in bed so that they can communicate more easily with each other. This is the only thing that can be done to provide comfort for them.” (Nurse 3).

“Family members in bereavement period before death are such that they accept everything we tell them and do not allow an unprofessional person from their friends or family to get too close to them. short our words are valid for them and, they like us to accompany them” (Nurse 8).

#### Contact With Other Bereaved Families

Participants believed that having communication with families who have similar conditions in the bereavement period is efficient and comforting.

“We often find the contact numbers of families whose children have died of cancer, and we tell bereaved families to call them and see how they cope. You do not believe that we often find out that families are together, Befriending and helping each other to get back to a normal life and even think about getting pregnant again.” (Nurse 6).

“I wish that conditions would be created in the hospital for families who are in the period of bereavement to come together and talk to each other. Do you know that these families are ashamed to communicate with those around them because of their childish illness. Cancer It is a taboo for them. But if they see a family with the same conditions, their suffering may be reduced”…(Nurse 11).

#### Support in Passing and Accepting the Bereaved

Support from family, social resources, and health care personnel can be efficient in overcoming the bereavement phase and making it easier to accept grief, as well as reducing the burden of bereavement on the family.

“We have to know if the bereavement keeps the family away from those around or not. Can the family do other children's chores to go to school? Or is his work messed up? Therefore, the family needs support in the bereavement period to meet all its needs.” (Nurse 5).

“A patient who has died, we set up with colleagues to go to the grave of the child in groups. Then their families even go to the ward 6, 7, 10, and 11 months later and appreciate how good it was. We went through a difficult time, but your memories are still in our minds” (Nurse 15).

#### Continuing Empathetic Communication With the Family, the Category

According to nurses' experience, establishing a good relationship with the family after the child's death can make the acceptance of death easier for the family.

“There are many cases where the personnel have communication with the families after the child's death. In some cases even if the patient died 2 years ago, but the mother still calls in a state of denial and says, Tell me what happened that night. This connection can help the family to accept the death of their child.” (Nurse 7).

“Personally, I cannot communicate with them afterwards. Now a series of colleagues call and talk, now they ask, but I cannot communicate at all, after this happens and goes away, I want to call and ask how he is, I cannot. I can't do anything with myself. I cannot at all. After this case, I can no longer communicate with my mother. ‘It 's as if I do not know what to say to them,' which is why they're cut off.” (Nurse 4).

### Category 2: Abandoned Family Access to Care

This category emphasizes the abandonment of the family after the child's death. According to the participants' opinion, after the child's death, the family is abandoned, and no care is provided. This category includes “improving family self-control” and “Family Awareness of End-of-Life Care.”

#### Promotion of Family Self-Control

Family self-control involves professional communication with the family so that the family retains or acquires a sense of control over the situation.

“After child's death, families are practically abandoned. A noticeable thing that they do is supporting the family after death, for example, they called the father of the family, and he said that ‘Come here, I need you.' They talk to them psychologically and calm them down.” (Nurse 7).

“Families are confused in this situation and do not perform many of their roles in the home and community properly. In these cases, it is better to give families specific responsibilities and the nurse has the role of counselor in empowering the family.” (Nurse 4).

#### Awareness of End-of-Life Care to the Family

Participants stated that families in the bereavement period need to obtain information and training from the treatment team on pre-and post-bereavement condition management and various end-of-life counseling. Most families had no proper method to control bereavement before and after the child's death.

“For example, I have taught many times, and I say that ‘well, try to be calm and trust in God and do not cry too much, because your child hears your voice even in the last moment of his life' after a while, they continue the same routine as before, and there is no change. There must be a training program that is taken seriously and they can be influenced by it.” (Nurse 6).

“It should be a training program that is done seriously and families can be affected by it. It seems that the training we are giving seems to have no effect. Now I do not know, maybe it is because of the little time we spend on them that I tell you, or because of the problems, or because it is too busy, I do not know, maybe for example the training should be from Be someone else who has an impact” (Nurse 8).

### Category 3: Continuing Care

The development of a child with cancer is stressful. Family involvement in the disease and treatment process leads to the reduction of resistance to problems. This can imbalances in life and the need for care throughout diagnosing process of a child with cancer until the post-bereavement stages. This category includes “formal and informal family care” and “individualized care.”

#### Formal and Informal Family Care

Participants believed that it was necessary to provide formal care by health care professionals, including physicians and nurses, as well as informal care by family caregivers in the face of the bereavement process.

“It is true that we do things for the family in the hospital which may calm them down a bit. On the other hand, the main stage of their adaptation to this situation is when they go home and need professional people to be with them.” (Nurse 12).

“Many times, moms say that they call Famila to ask how we are, but now that they do not ask, they all get sore. Hey, they say, look what you have done, God has made you like this. Or they say, every time they call, they say, ‘How are you?' The disease is the same. I mean, in fact, there are a series of disorders that I feel are bothering the family and there is no informal support for them, so official health caregivers should provide support at home and in the personal life of the bereaved family” (Nurse3).

#### Individualized Care

Preparing for death, considering the specific needs of each child and family, and post-bereavement care are integral parts of care.

“Families are very different from each other, one is rich, one is religious, and in short, they are different, and you do not know how to behave. For example, 1 day, I told a mother that there are charities that can help the families suddenly, the mother got angry and said, “Are we begging?” (Nurse 9).

“The wishes of the bereaved families may be similar in general, but they may differ from person to person. There must be an expert who knows what to say through which door to enter and how to guide them, because they really say something that only A team of experts can understand their needs from what they say” (Nurse 12).

## Discussion

In this study, the bereavement needs of families who have a child with cancer were explained from the perspective of health caregiver. The needs of bereaved families include the three categories of “achieving peace,” “Abandoned family access to care,” and “continuing care.”

Regarding the category of achieving peace, based on the experience of the participants, during the bereavement period, the internal management of the house suffers, and the family becomes paralyzed. Following the pressures of illness and treatment, the strength of the family weakens, and the process of life becomes difficult. So that in the face of this crisis, they are constantly asking for help and support to maintain and strengthen their cohesion. The reflection of needing help is the need for spiritual and existential support, companionship with the family, contact with other bereaved families, support in passing and accepting the bereaved and continuing empathetic communication with the family. Many participants believed in calming the patient and family in the end of life and considered proper communication, as well as the cessation of painful aggression. Spiritual care supports a sense of hope, value, the purpose of connecting with others, and connecting to greater power in the child and family. Some participants believed that the remembrance of God and religious practices heal to reduce the child's pain and illness, but the possibility and support are not provided in this regard. It seems that spiritual care for parents during a child's illness is necessary because they look to help in the body of religion. Nikseresht et al. (2016) also recommended the use of spiritual and religious approaches as an efficient way to deal with the stresses caused by cancer in Iranian society. Rassouli et al. also considered spiritual needs as one of the most important needs of Iranian cancer patients and their families (Rassouli and Sajjadi, 2014). In line with this, the results of a qualitative study showed that religion has known as the main theme of the supportive factor for pediatric cancer adaptation (Stanton et al., 2015).

Regarding companionship with the family, it can be said that almost all participants in the study emphasized the effect of health caregivers' behavior on increasing or decreasing the morale of the child and family. Esbensen et al. (2008) suggested that professional staff spend more time listening to patients and paying attention to their interests. But Support from professional staff has been shown to be necessary only when the family network is dysfunctional, with poor communication (Näppä et al., 2016). A review study by Laura et al. showed that one of the basic needs of families was caregiver communication skills with the family. In consist with the results of the present study, effective communication and method of telling the truth can have a positive outlook for the family (Kerr et al., 2004).

In the subcategory of contact with other bereaved families, participants believed that during the bereavement period, contact and communication with families with similar conditions are effective and comforting. However, during this period, some communications are strengthened, and some are reduced. In addition, aspects of family communication such as sharing feelings, appreciating each other, listening, and expression skills are important, positive, and supportive. The bereavement groups seemed to produce positive effects which could not be captured by the chosen outcome measures, such as a deeper insight into the grieving process and a feeling of joint experience in grief. It cannot be told how anxiety, depressive mood and grief would have evolved in participants without the bereavement intervention. The results of Nappa study indicate that we as health care staff do not have to worry about most of those persons who decline participation in bereavement groups, as they show less severe grief and anxiety than others (Näppä et al., 2016).

Support in passing and accepting the bereaved was another subcategory of achieving peace. The dissolution of the attachment relationship with the child causes severe anxiety and other negative emotions in parents. Parents may feel guilty about not being able to protect their children. Research emphasizes that bereavement is an injury that causes negative psychological and health effects (Rogers et al., 2008). Stroebe's study found that bereaved were more likely to commit suicide (Stroebe et al., 2005). Li et al. (2005) that the bereaved were at risk of psychiatric hospitalization, especially the mother. The risk of a mother's hospitalization may continue for more than 5 years after the child's death (Candy et al., 2015). However, Lindemann stated in the 1940s that if normal grief is shared with professional help, it is possible to settle an uncomplicated grief reaction in 4–6 weeks (Näppä et al., 2016). Danish researchers have found that the death rate of bereaved parents is higher than that of non-bereaved parents. Thus, bereavement harms social functioning and quality of family life (Li et al., 2003). Families bereaved by cancer also often struggle with isolation due to fear of burdening their support network with their persistent pain (Hinds et al., 1997) and experience decreased social support over time (Lichtenthal et al., 2015).

Regarding continuing empathetic communication with the family, it can be said that according to the participants, after the child's death, the families are practically abandoned and, no care is provided. Some health caregivers make limited phone calls to bereaved families without written instructions. In a study, the satisfaction and uniqueness of the role of volunteers in care, from the perspective of the patient and family have been mentioned (Candy et al., 2015). According to the cultural and religious context of the country and the existence of numerous charities, it can be adapted to the prevailing condition (Rassouli and Sajjadi, 2014). While there is no right or wrong way to child loss bereavement, the Australian Care Program helps families know which response is right or wrong and helps families to identify when family members need counseling and support (Palliative Care Australia, 2010). In Canada, research has shown that parents are constantly in need of bereavement services as part of their care. There is a wide variety of bereavement programs where services can include a range of telephone or letter calling programs, grief support groups (siblings or parents), or meetings with professionals to discuss the death of a child or autopsy results (Widger et al., 2012).

The second category was abandoned family access to care. Regarding the sub-category promotion of family self-control and awareness of end-of-life care to the family, participants acknowledged that despite providing care, factors such as child and family wandering in different areas of care, insufficient knowledge of medical resources, and spending too much time accessing services. The family faces problems that require coordination between different levels of treatment to receive ongoing care. Consistent with the present study, a study aimed at assessing the information and health needs of immigrants with cancer showed that the most information needs of patients were access to information about healthier living and disease control and management, including information needs related to nutrition, physical activity and pharmaceutics information (Riahi et al., 2016). Parents like to be involved in treatment decisions and know the risks and side effects of treatment. These cases are consistent with the results of the present study. It can be said that having information about the child's condition is essential for parents' peace of mind, feeling of being in control of the situation, staying optimistic and developing strategies that are in the best interest of the child (Kerr et al., 2004). In line with the findings of the present study, in a needs assessment in parents of children with cancer, information needs were recognized as a common need (Kerr et al., 2004). Moreover, a study showed that parents highly rated involvement in decision making regarding care and treatment of their child, although decision making during the palliative phase is acknowledged as being extremely difficult for parents (Hinds et al., 1997). In addition, although parents perceive their decisions around their child's care and treatment needs as representative of their child's needs (Van der Geest et al., 2014). There is evidence that parents' understanding about their child's prognosis may not always be realistic (Wolfe et al., 2000). Therefore, involvement and support of health care professionals remain crucial (Van der Geest et al., 2014).

The last category of this study was called continuing care. Formal and informal family care should be based on an understanding of the position of the family and the child in the family network, friends and community, interactions, and communication. Findings showed that having good relationships and sources of support from family and those around are factors in reducing the burden of disease on the child and family, and the absence of these positive factors harms the child and family. Social care is defined as the provision of people whom a person trusts and feels respected. This type of support in stressful situations such as cancer diagnosis is considered an important source of psychological support (Candy et al., 2015). Despite families' risk for poor outcomes, few bereavement follow-up programs and formal interventions to support them have been rigorously evaluated. A systematic review of studies on hospital-based bereavement programs concluded that such programs help families feel cared for, reduce their sense of isolation, and improve their coping (Donovan et al., 2015). The results of a study showed parents were positive about the collaboration between the tertiary care, local hospital, and community health care professionals and highly rated the frequency of consultations with health care professionals from the hospital in the period before their child died, in the time period between their child's death and funeral, and in the period thereafter (Van der Geest et al., 2014).

Regarding continuity of care, earlier reported benefits of continuity of care, for instance, reducing parental frustration and enhancing parents' confidence in quality of care could clarify why continuity of care during the palliative phase is related to lower levels of parents' long-term grief. In contrast with communication and continuity of care, Van der Geets study showed that the extent to which parents felt involved by health care professionals turned out not to be related with parental grief levels (Van der Geest et al., 2014).

Children and adolescents are in the midst of the process of physical, emotional, cognitive, and spiritual development and have different needs depending on their stage of development, and their care should be individualized. Their families also communicate in different ways, and their understanding of illness and death depends on their child's stage of development and experience. Therefore, many participants considered that family care based on their child's development is necessary. However, research findings indicate that the child's anxiety about hospitalization is closely related to parents' mental states. Parents' tendency to behave abnormally with a sick child, giving the child a special position, and being vulnerable has negative consequences and leads to the child's secondary mental disability.

## Conclusion

Dedication to providing excellent care during treatment and into bereavement for seriously ill children and their family members is crucial for holistic, patient-, and family entered care. Parental grief is complex and support for bereaved parents is universally needed, albeit with individual differences (Spraker-Perlman et al., 2021). According to the challenges and needs of the family before and after the death of the child, the care team needs to pay special attention to the families of the bereaved patients. Therefore, it is recommended that members of the health care team be trained in assessing family needs, identifying risks from adverse outcomes, continuing care, and providing resources during bereavement. The needs of the bereaved family should also be addressed in their care program. The results of the present study can provide broader bereavement care, but more studies are needed to clarify other experiences and families' perceptions of the needs of the bereavement period. Limitations of this study included the impossibility of generalizing the results and the lack of cooperation of bereaved families to participate in the study.

## Data Availability Statement

The raw data supporting the conclusions of this article will be made available by the authors, without undue reservation.

## Author Contributions

MP: investigation, wrote the manuscript, and writing—review and editing. MR: conceptualization methodology and writing—review and editing. NR, SR, and SB: writing—review and editing. SM: project administration, conceptualization, methodology, and supervision. All authors contributed to the article and approved the submitted version.

## Conflict of Interest

The authors declare that the research was conducted in the absence of any commercial or financial relationships that could be construed as a potential conflict of interest.

## Publisher's Note

All claims expressed in this article are solely those of the authors and do not necessarily represent those of their affiliated organizations, or those of the publisher, the editors and the reviewers. Any product that may be evaluated in this article, or claim that may be made by its manufacturer, is not guaranteed or endorsed by the publisher.
